# Supplementary material for: Hidden and Understaffed: Exploring Canadian Medical Laboratory Technologists’ Pandemic Stressors and Lessons Learned
Source: Healthcare (Basel). 2023 Oct 14;11(20):2736. doi: 10.3390/healthcare11202736 (PMC10606905; doi:10.3390/healthcare11202736)
Supplement: Supplementary file 1 [file healthcare-11-02736-s001.zip › healthcare-2641429-supplementary.pdf]

Section S1: Interview Guide

Before the focus group:

Researchers establish rapport with the participants. Participants are reminded of the study's purpose, risks, benefits, confidentiality of information, and anonymity. The moderator reminds participants that the session will be audio recorded to ensure accuracy of data.

Questions:

1. Can you discuss the primary job roles included in your position?
2. As a medical laboratory technologist, what are the common stressors within your profession?
    a. Follow-up questions as necessary.
3. What are the common stressors during the pandemic and what are the implications of these stressors on patient care?
    a. Follow-up questions as necessary.
4. What are some non-work factors that contributed to stress at work during the pandemic?
    a. Follow-up questions as necessary.
5. Describe the lessons learned from the COVID-19 pandemic that could help reduce stress in the event of future pandemics.
    a. Follow-up questions as necessary.
6. From the lens of your profession and your experiences during the pandemic, what can be done to help optimize the healthcare system?
    a. Follow-up questions as necessary.
7. Is there anything else you would like to add?

After the focus group:

Receive feedback from participants

Section S2: COREQ

Item No.	Item	Guide questions/description	
**Domain 1: Research team and reflexivity**			
Personal characteristics			
1	Interviewer	Which author/s conducted the interview or focus group?	Myuri Sivanthan, Basem Gohar, Patricia Nicole Dignos, Michael Gardiner-Davis
2	Credentials	What were the researcher's credentials?	Myuri Sivanthan: Ph.D., M.H.Sc.<br>Basem Gohar: Ph.D., M.Sc., C. Psych.<br>Patricia Nicole Dignos: H.B.Sc.<br>Michael Gardiner-Davis: H.B.Sc.
3	Occupation	What was their occupation at the time of the study?	Myuri Sivanthan: Post-doctoral fellow<br>Basem Gohar: Assistant Professor & Clinical Psychologist<br>Patricia Nicole Dignos: Graduate student<br>Michael Gardiner-Davis: Undergraduate student
4	Gender	Was the researcher male or female?	Myuri Sivanthan: Female<br>Basem Gohar: Male<br>Patricia Nicole Dignos: Female<br>Michael Gardiner-Davis: Male
5	Experience and training	What experience or training did the researcher have?	Basem Gohar has extensive experience on qualitative research and working with various healthcare providers, including medical laboratory professionals. Myuri Sivanthan has research experience with various healthcare providers.
Relationship with participants			
6	Relationship established	Was a relationship established prior to study commencement?	Researchers were in contact with the participants via email correspondence. Prior to the start of the study, the researchers introduced themselves to the participants. Participants also had a chance to introduce themselves to the focus group.
7	Participant knowledge of the interviewer	What did the participants know about the researcher?	Participants were informed about the researchers' research interests and experience. They were also informed of the study's purpose.

8	Interviewer characteristics	What characteristics were reported about the interviewer/facilitator?	One of the facilitators (B.G.) previously conducted research on medical laboratory professionals at the provincial level. Open-ended questions during the focus group allowed participants to describe their experiences without bias from the interviewers/facilitators.
**Domain 2: Study Design**			
Theoretical Framework			
9	Methodological orientation & theory	What methodological orientation was stated to underpin the study?	Qualitative descriptive study
Participant selection			
10	Sampling	How were participants selected?	Participants were selected in partnership with various associations and regulatory bodies across Canada, using social media platforms, and snowball sampling technique.
11	Method of approach	How were participants approached?	Participants were approached by email.
12	Sample size	How many participants were in the study?	A total of 27 participants were in the study.
13	Non-participation	How many people refused to participate or dropped out? Reasons?	Among the interested participants, six dropped out.
Setting			
14	Setting of data collection	Where was the data collected?	Online via Microsoft Teams
15	Presence of non-participants	Was anyone else present besides the participants and researchers?	No
16	Description of sample	What are the important characteristics of the sample?	See Table 1.
Data collection			
17	Interview guide	Were questions, prompts, guides provided by the authors? Was it pilot tested?	The questions were prompted by the authors. The questions were adopted from a previous study.
18	Repeat interviews	Were repeat interviews carried out? If yes, how many?	No, focus groups were not repeated.

19	Audio/visual recording	Did the research use audio or visual recording to collect the data?	The research used audio recording to collect the data
20	Field notes	Were field notes made during and/or after the interview or focus group?	Yes
21	Duration	What was the duration of the interviews or focus group?	The focus groups were approximately 90 minutes.
22	Data saturation	Was data saturation discussed?	Yes, data saturation was reached after 5 focus groups.
23	Transcripts returned	Were transcripts returned to participants for comment and/or correction?	Transcripts were not returned to participants. However, as a form of member-checking, researchers paraphrased participants' responses during the focus group to ensure accuracy of data collection.
**Domain 3: Analysis and Findings**			
Data analysis			
24	Number of data coders	How many data coders coded the data?	Two data coders coded the data.
25	Description of the coding tree	Did authors provide a description of the coding tree?	Yes. See table 2.
26	Derivation of themes	Were themes identified in advance or derived from the data?	Yes
27	Software	What software, if applicable, was used to manage the data?	Quirkos
28	Participant checking	Did participants provide feedback on the findings?	No
Reporting			
29	Quotations presented	Were participant quotations presented to illustrate the themes/findings? Was each quotation identified?	Yes
30	Data and findings consistent	Was there consistency between the data presented and the findings?	Yes

31	Clarity of major themes	Were major themes clearly presented in the findings?	Yes
32	Clarity of major themes	Is there a description of diverse cases or discussion of minor themes?	Yes
